# Prognostic Value of HALP Score in Rheumatic Mitral Stenosis: A Long‐Term Follow‐up Study

**DOI:** 10.1002/clc.70287

**Published:** 2026-03-27

**Authors:** Ahmet Ferhat Kaya, Görkem Ayhan, Veysi Can, Emrah Özbek, Lezgin Dursun, Ömer Kümet

**Affiliations:** ^1^ Department of Cardiology Van Regional Education and Research Hospital Van Turkey; ^2^ Department of Cardiology Bingöl State Hospital Bingöl Turkey

**Keywords:** HALP score, inflammation, major adverse cardiac and cerebrovascular events (MACCE), nutrition, prognosis, rheumatic mitral stenosis, risk stratification

## Abstract

**Objective:**

This study aimed to evaluate the prognostic value of the hemoglobin–albumin–lymphocyte–platelet (HALP) score in patients with rheumatic mitral stenosis (RMS).

**Methods:**

A total of 169 patients with RMS were retrospectively analyzed over a median follow‐up period of 124 months (IQR: 96–148). The primary endpoint was major adverse cardiac and cerebrovascular events (MACCE). The predictive performance of the HALP score was assessed using receiver operating characteristic (ROC) analysis, Cox regression, and Kaplan–Meier survival analysis.

**Results:**

The HALP score demonstrated a modest but statistically significant ability to predict MACCE (AUC = 0.644, *p* = 0.037). The optimal cut‐off value was 35.65, with a sensitivity of 60.4% and specificity of 38.1%. Patients with lower HALP scores had a significantly higher incidence of MACCE (log‐rank *p* = 0.015). However, in multivariable analysis, the HALP score was not identified as an independent predictor (*p* = 0.076).

**Conclusion:**

The HALP score may serve as a simple and cost‐effective complementary tool for risk stratification in patients with RMS. However, its prognostic value appears limited when used alone, and it should be interpreted alongside established clinical and echocardiographic parameters.

## Graphical abstract

In 169 patients with rheumatic mitral stenosis, a low HALP score was associated with increased long‐term MACCE risk. The HALP score showed modest predictive value (AUC 0.644) but was not an independent predictor. It may serve as a simple complementary tool for risk stratification alongside established clinical parameters.

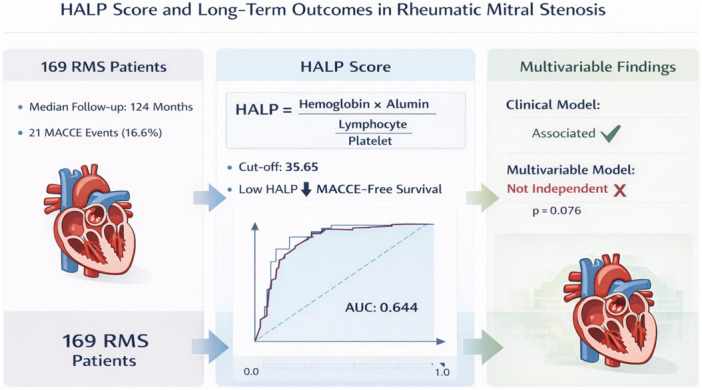

## 1 Introduction

Rheumatic valve disease is characterized by progressive valvular damage resulting from autoimmune inflammation following acute rheumatic fever (ARF) caused by group A beta‐hemolytic streptococci (GAS) [1]. Antibodies developed against GAS exhibit similarity to epitopes present in cardiac valve tissues, leading to a cross‐reaction known as molecular mimicry [2]. This immune‐mediated process results in inflammation, fibrosis, and structural remodeling of the valvular apparatus, ultimately leading to valvular stenosis and/or regurgitation [2].

Rheumatic mitral stenosis (RMS) is the most common form of acquired valvular heart disease worldwide and remains an important cause of morbidity and mortality, particularly in developing countries [3, 4, 5]. Early identification of high‐risk patients is crucial for optimizing management and improving long‐term outcomes. Therefore, simple, reliable, and cost‐effective biomarkers are of great clinical interest.

The hemoglobin–albumin–lymphocyte–platelet (HALP) score is a composite biomarker that integrates inflammatory, nutritional, and hematological parameters. Initially introduced as a prognostic marker in oncology, it has recently been investigated in cardiovascular diseases [3]. The HALP score is calculated as follows: HALP = (hemoglobin × albumin × lymphocyte count) ÷ platelet count [4].

Despite increasing interest in the HALP score, its prognostic value in patients with rheumatic mitral stenosis has not been well established. Given that RMS is characterized by chronic inflammation and systemic involvement, the HALP score may provide a comprehensive reflection of disease burden.

To our knowledge, limited data exist regarding the long‐term prognostic role of the HALP score in RMS. Therefore, this study aimed to investigate the association between the HALP score and long‐term major adverse cardiac and cerebrovascular events (MACCE) in patients with rheumatic mitral stenosis.

## 2 Materials and Methods

### 2.1 Study Population

This retrospective observational study included patients diagnosed with rheumatic mitral stenosis (RMS) who were followed at our institution between January 2014 and December 2019. Patients with active infection, malignancy, chronic kidney disease, other rheumatologic diseases, prior stroke, peripheral arterial disease, or pregnancy were excluded.

### 2.2 Data Collection

Demographic and clinical data were obtained from hospital records and outpatient follow‐up files. Collected variables included age, sex, comorbidities (hypertension, diabetes mellitus, and hyperlipidemia), smoking status, medication use, and echocardiographic findings.

### 2.3 Laboratory Analysis

Laboratory parameters included hemoglobin, albumin, lymphocyte count, platelet count, complete blood count, biochemical parameters, and inflammatory markers such as C‐reactive protein (CRP). These parameters were used to calculate the HALP score and for additional analyses [3].

### 2.4 Echocardiographic Assessment

Transthoracic echocardiography was performed in accordance with the guidelines of the American Society of Echocardiography (ASE) [6]. All measurements were performed by two experienced cardiologists blinded to clinical outcomes. Evaluated parameters included left atrial diameter, left ventricular end‐diastolic diameter, left ventricular ejection fraction, mitral valve area (using planimetric and pressure half‐time methods), transmitral gradients, and associated valvular involvement. Systolic pulmonary artery pressure was calculated from the tricuspid regurgitation jet.

### 2.5 HALP Score Calculation

The HALP score was calculated using the following formula:

HALP = (hemoglobin × albumin × lymphocyte count) ÷ platelet count [4].

### 2.6 Follow‐Up and Clinical Outcomes

Patients were followed for a median duration of 124 months (IQR: 96–148). The primary endpoint was major adverse cardiac and cerebrovascular events (MACCE), including death, myocardial infarction, stroke, and mitral valve interventions. Mitral valve interventions (percutaneous mitral balloon valvuloplasty or surgery) were included as indicators of disease progression and clinical deterioration.

### 2.7 Statistical Analysis

Statistical analyses were performed using SPSS version 25.0 (IBM Corp., Armonk, NY, USA). The normality of continuous variables was assessed using the Shapiro–Wilk and Lilliefors tests. Normally distributed variables were expressed as mean ± standard deviation (SD), whereas non‐normally distributed variables were presented as median and interquartile range (IQR). Categorical variables were expressed as frequencies and percentages.

Comparisons between groups were performed using the Student's *t*‐test or Mann–Whitney *U* test for continuous variables, and the chi‐square test or Fisher's exact test for categorical variables.

Receiver operating characteristic (ROC) curve analysis was used to evaluate the predictive performance of the HALP score for MACCE, and the area under the curve (AUC) was calculated. The optimal cut‐off value was determined using the Youden index. The HALP score was also included as a continuous variable in Cox proportional hazards regression analysis to assess its independent predictive value.

Survival analyses were performed using the Kaplan–Meier method, and patients were categorized into low and high HALP groups based on the ROC‐derived cut‐off value. A two‐sided *p*‐value < 0.05 was considered statistically significant.

## 3 Results

A total of 169 patients diagnosed with rheumatic mitral stenosis (RMS) were included in the study. The baseline demographic and clinical characteristics of the study population are presented in Table 1. There was a marked female predominance (81.9%), and the median age was 41 years. Comorbid conditions such as hypertension (14.2%), diabetes mellitus (10.2%), and hyperlipidemia (13.4%) were relatively infrequent. Atrial fibrillation was present in 30.7% of patients. The majority of patients were receiving beta‐blocker therapy (95.3%). During follow‐up, 27.6% of patients underwent percutaneous mitral balloon valvuloplasty (Table 1).

**Table 1:** Demographic and clinical characteristics of the study population.

Variable	All patients (*n* = 169)
Sex, *n* (%)	
Female	138 (81.9)
Male	31 (18.1)
Age, years	41 ± 11.8
Hyperlipidemia, *n* (%)	17 (13.4)
Diabetes mellitus, *n* (%)	13 (10.2)
Hypertension, *n* (%)	18 (14.2)
Chronic kidney disease, *n* (%)	3 (1.8)
Cardiac rhythm, *n* (%)	
Atrial fibrillation	39 (30.7)
Sinus rhythm	88 (69.3)
Smoking, *n* (%)	37 (29.1)
MACCE, *n* (%)	
None	106 (83.5)
Myocardial infarction	1 (0.8)
Cerebrovascular event	3 (2.4)
Death	17 (13.4)
Percutaneous mitral balloon valvuloplasty, *n* (%)	35 (27.6)
Surgery, *n* (%)	23 (18.1)
Follow‐up duration, months	124 (96–148)
Medications, *n* (%)	
Beta‐blockers	121 (95.3)
ACE inhibitors/ARBs	13 (11.0)
Mineralocorticoid receptor antagonists	15 (11.8)
Oral anticoagulants	21 (16.5)
Diuretics	68 (53.5)

Abbreviations: ACE, angiotensin‐converting enzyme; ARB, angiotensin receptor blocker; MACCE, major adverse cardiac and cerebrovascular events.

During the follow‐up period, the overall incidence of major adverse cardiac and cerebrovascular events (MACCE) was 16.6%, including death (13.4%), myocardial infarction (0.8%), and stroke (2.4%).

Laboratory findings are summarized in Table 2. The median C‐reactive protein (CRP) level was 0.8 mg/dL (IQR: 0.4–1.2), indicating the presence of low‐grade chronic inflammation. The median albumin level was 3.6 g/dL (IQR: 3.3–4.0), and the mean hemoglobin level was 12.5 ± 1.7 g/dL. These findings suggest a mild impairment in nutritional and inflammatory status. Platelet count and white blood cell (WBC) subtypes were within normal ranges (Table 2).

**Table 2:** Laboratory findings.

Laboratory findings	All patients (*n* = 169)
Glucose (mg/dL)	101 (91–115)
Urea (mg/dL)	27 (21–34)
Creatinine (mg/dL)	0.7 (0.6–0.8)
Sodium (mEq/L)	137 (135–139)
Potassium (mEq/L)	4.2 (4.0–4.5)
Calcium (mg/dL)	9.0 (8.5–9.5)
C‐reactive protein (mg/dL)	0.8 (0.4–1.2)
LDH (U/L)	276.9 ± 104.4
Platelet (10³/mm³)	255.6 ± 93.7
MPV (fL)	8.9 ± 1.8
Albumin (g/dL)	3.6 (3.3–4.0)
Hemoglobin (g/dL)	12.5 ± 1.7
Hematocrit (%)	38.4 ± 5.0
MCV (fL)	84.0 (80.0–87.0)
MCH (pg)	28.0 (26.0–29.0)
MCHC (g/dL)	33.0 (32.0–34.0)
RDW (%)	13.8 ± 2.4
AST (IU/L)	22.0 (17.0–28.0)
ALT (IU/L)	17.0 (12.0–25.0)
WBC (×10³/µL)	9.0 (7.5–10.1)
RBC (×10⁶/µL)	4.5 ± 0.6
Neutrophils (×10⁹/L)	5.6 (4.4–7.4)
Lymphocytes (×10⁹/L)	2.1 (1.6–2.6)
Monocytes (×10⁹/L)	0.5 (0.4–0.7)
Basophils (%)	0.07 (0.05–0.10)
Eosinophils (%)	0.09 (0.06–0.17)

*Note:* Values are presented as median (interquartile range) or mean ± standard deviation, as appropriate.

Echocardiographic parameters are presented in Table 3. The median left atrial diameter was 46 mm (IQR: 43–51), indicating left atrial enlargement. The median left ventricular ejection fraction was preserved at 60%. The mitral valve area was 1.3 cm² (IQR: 1.0–1.4), as assessed by both planimetric and pressure half‐time methods. Severe mitral stenosis (valve area < 1 cm²) was present in 31.5% of patients, most of whom subsequently underwent surgical intervention. Moderate to severe tricuspid regurgitation was commonly observed, and the median systolic pulmonary artery pressure was 40 mmHg (IQR: 30–50), indicating mild pulmonary hypertension (Table 3).

**Table 3:** Echocardiographic parameters.

Echocardiographic parameters	All patients (*n* = 169)
Ejection fraction (%)	60 (60–60)
Left atrial diameter (mm)	46 (43–51)
Interventricular septum (mm)	10.0 (9.0–11.0)
Left ventricular end‐diastolic diameter (mm)	46.0 ± 4.7
Mitral valve area (PHT, cm²)	1.30 (1.0–1.4)
Mitral valve area (planimetry, cm²)	1.30 (1.0–1.4)
Mean gradient (mmHg)	10.0 (8.0–13.0)
Maximum gradient (mmHg)	20.0 (16.0–25.0)
Mitral stenosis severity—None	0 (0)
Mitral stenosis severity—Mild	0 (0)
Mitral stenosis severity—Moderate	87 (68.5)
Mitral stenosis severity—Severe	40 (31.5)
Two‐valve involvement	57 (44.9)
Aortic regurgitation—None	70 (55.1)
Aortic regurgitation—Mild	42 (33.1)
Aortic regurgitation—Moderate	14 (11.0)
Aortic regurgitation—Severe	1 (0.8)
Mitral regurgitation—None	49 (38.6)
Mitral regurgitation—Mild	51 (40.2)
Mitral regurgitation—Moderate	25 (19.7)
Mitral regurgitation—Severe	2 (1.6)
Tricuspid regurgitation—None	11 (8.7)
Tricuspid regurgitation—Mild	80 (63.0)
Tricuspid regurgitation—Moderate	29 (22.8)
Tricuspid regurgitation—Severe	7 (5.5)
Systolic pulmonary artery pressure (mmHg)	40.0 (30.0–50.0)

*Note:* Values are presented as median (interquartile range) or mean ± standard deviation, as appropriate.

Abbreviations: PHT, pressure half‐time.

**Figure 1:**
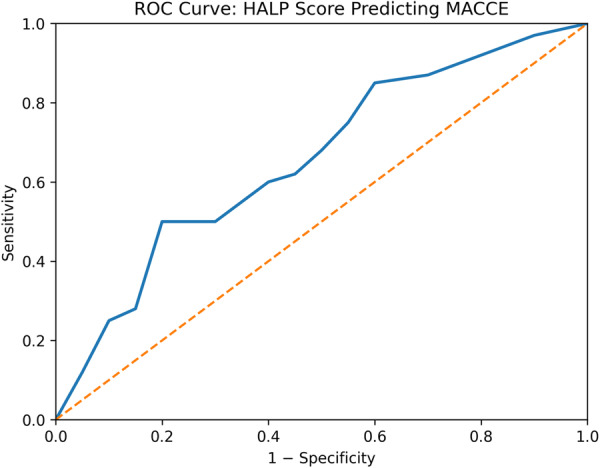
Receiver operating characteristic (ROC) curve analysis of the HALP score for predicting major adverse cardiac and cerebrovascular events (MACCE).

Receiver operating characteristic (ROC) analysis demonstrated that the HALP score had a modest but statistically significant ability to predict MACCE (AUC = 0.644; 95% CI: 0.512–0.777; *p* = 0.037). The optimal cut‐off value was 35.65, with a sensitivity of 60.4% and specificity of 38.1% (Table 4, Figure 1).

**Table 4:** Receiver operating characteristic analysis of the HALP score for predicting MACCE.

Variable	AUC (95% CI)	Cut‐off	*p* value	Sensitivity (%)	Specificity (%)
HALP score	0.644 (0.512–0.777)	35.65	0.037	60.4	38.1

Abbreviations: AUC, area under the curve; CI, confidence interval; MACCE, major adverse cardiac and cerebrovascular events.

In Cox regression analysis, the HALP score showed a borderline association with MACCE but did not reach statistical significance (OR = 0.976; 95% CI: 0.950–1.003; *p* = 0.076) (Table 5).

**Table 5:** Cox regression analysis of predictors of MACCE.

Variable	Odds ratio (OR)	95% Confidence interval	*p* value
HALP score	0.976	0.950–1.003	0.076

Abbreviations: CI, confidence interval; MACCE, major adverse cardiac and cerebrovascular events; OR, odds ratio.

**Figure 2:**
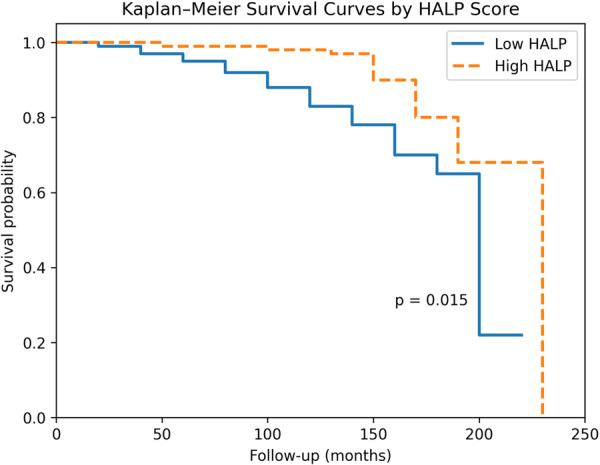
Kaplan–Meier survival curves according to HALP score for predicting major adverse cardiac and cerebrovascular events (MACCE).

Kaplan–Meier survival analysis demonstrated that patients with low HALP scores had a significantly higher incidence of MACCE compared to those with high HALP scores (log‐rank *p* = 0.015) (Figure 2).

## 4 Discussion

In this study, we evaluated the long‐term prognostic value of the HALP score in patients with rheumatic mitral stenosis (RMS). Our findings demonstrated that a low HALP score was significantly associated with an increased incidence of major adverse cardiac and cerebrovascular events (MACCE). Kaplan–Meier analysis revealed a significant difference in event‐free survival between groups, whereas the association did not reach statistical significance in multivariable Cox regression analysis. These results suggest that the HALP score may serve as a complementary rather than a standalone prognostic marker.

The HALP score is a composite index reflecting multiple biological pathways, including inflammation, immune response, and nutritional status. Hemoglobin levels may reflect chronic disease burden and oxygen‐carrying capacity, albumin levels are associated with both nutritional status and systemic inflammation, lymphocyte count reflects immune competence, and platelet count is linked to pro‐inflammatory and prothrombotic activity. By integrating these parameters, the HALP score provides a more comprehensive assessment of the patient's overall condition.

Previous studies have demonstrated the prognostic significance of the HALP score in various clinical settings. In oncology, it has been associated with survival outcomes [7], while in cardiovascular diseases, low HALP scores have been linked to increased mortality in acute coronary syndromes [3], heart failure [8], and valvular heart disease [9]. Additionally, nutritional indices have been shown to correlate with adverse outcomes in patients with RMS [10]. Our findings extend this evidence by demonstrating a similar association in patients with rheumatic mitral stenosis.

Rheumatic mitral stenosis is characterized by a chronic inflammatory and fibrotic process in which immune‐mediated mechanisms play a central role [1, 2, 5]. In this context, a composite index such as the HALP score, which captures both inflammatory and nutritional status, is biologically plausible as a prognostic marker. However, the relatively modest predictive performance observed in our study (AUC = 0.644) indicates that the HALP score alone is insufficient for accurate risk stratification.

Compared to other inflammatory markers such as the neutrophil‐to‐lymphocyte ratio (NLR) and platelet‐to‐lymphocyte ratio (PLR) [11, 12, 13], the HALP score has the advantage of incorporating nutritional parameters in addition to inflammatory components. This integrative approach may provide a broader reflection of patient status. Nevertheless, its incremental prognostic value appears limited, supporting its use as part of a multiparametric risk assessment rather than as an independent predictor.

From a clinical perspective, the HALP score is simple, cost‐effective, and readily available from routine laboratory parameters. This makes it particularly useful in resource‐limited settings, where advanced diagnostic tools may not be accessible. Incorporating the HALP score into existing risk models may improve the identification and monitoring of high‐risk patients.

### 4.1 Limitations

This study has several limitations. First, its retrospective and single‐center design may limit generalizability. Second, the relatively small sample size and limited number of events may have reduced statistical power and affected the stability of multivariable analyses. Third, the cut‐off value identified in this study requires validation in external cohorts. Finally, the HALP score was assessed at a single time point, and changes over time were not evaluated.

## 5 Conclusion

The HALP score provides a modest but statistically significant contribution to predicting long‐term outcomes in patients with rheumatic mitral stenosis. Although low HALP scores are associated with an increased risk of adverse events, the score should not be used as a standalone predictor. Instead, it should be incorporated into multiparametric risk models alongside established clinical and echocardiographic variables. Further prospective, multicenter studies are needed to validate these findings and to clarify the prognostic impact of longitudinal changes in the HALP score.

## Author Contributions

**Ahmet Ferhat Kaya:** conceptualization, writing. **Görkem Ayhan** and **Veysi Can:** data collection, writing. **Ahmet Ferhat Kaya** and **Emrah Özbek:** analysis, writing. **Ömer Kümet:** supervision, writing.

## Funding

The authors have nothing to report.

## Ethics Statement

This study was approved by the Non‐Interventional Clinical Research Ethics Committee of Van Training and Research Hospital (Decision No: GOKAEK/2025‐02‐06, Date: 28.02.2025) and conducted in accordance with the Declaration of Helsinki.

## Conflicts of Interest

The authors declare no conflicts of interest.

## Data Availability

The data supporting the findings of this study are available from the corresponding author upon reasonable request.

## References

[1] M. W. Cunningham , “Pathogenesis of Group A Streptococcal Infections,” Clinical Microbiology Reviews 13, no. 3 (2000): 470–511, 10.1128/CMR.13.3.470. 10885988 PMC88944

[2] L. Guilherme and J. Kalil , “Molecular Pathogenesis of Rheumatic Fever and Rheumatic Heart Disease,” Expert Review of Cardiovascular Therapy 3, no. 3 (2005): 345–352, 10.1586/14779072.3.3.345. 16336741

[3] H. Wang , Y. Zhang , W. Zhang , et al., “Hemoglobin, Albumin, Lymphocyte, and Platelet Score: A Novel Predictor of Mortality in Patients With Coronary Heart Disease,” Frontiers in Cardiovascular Medicine 10 (2023): 1241217, 10.3389/fcvm.2023.1241217. 38028472 PMC10679332

[4] T. Zhang , W. He , S. Jiang , et al., “HALP Score for Predicting Renal Relapse in Lupus Nephritis,” Journal of Clinical Rheumatology: Practical Reports on Rheumatic & Musculoskeletal Diseases (2024), 10.1097/RHU.0000000000002506. 40433955

[5] E. Marijon , M. Mirabel , D. S. Celermajer , and X. Jouven , “Rheumatic Heart Disease,” Lancet 379, no. 9819 (2012): 953–964, 10.1016/S0140-6736(11)61171-9. 22405798

[6] R. M. Lang , L. P. Badano , V. Mor‐Avi , et al., “Recommendations for Cardiac Chamber Quantification by Echocardiography in Adults: An Update from the American Society of Echocardiography and the European Association of Cardiovascular Imaging,” Journal of the American Society of Echocardiography 28, no. 1 (2015): 1–39.e14, 10.1016/j.echo.2014.10.003. 25559473

[7] X. Chen , X. Zhai , B. Zhao , et al., “HALP Score as a Novel Prognostic Indicator in Patients With Esophageal Squamous Cell Carcinoma,” Oncotarget 7, no. 39 (2016): 63568–63578, 10.18632/oncotarget.11574.

[8] L. Dai , Y. Liu , X. Zhang , et al., “Prognostic Impact of HALP Score in Patients With Heart Failure,” ESC Heart Failure 8, no. 6 (2021): 5240–5249, 10.1002/ehf2.13600.

[9] L. Liu , Y. Chen , H. Wang , et al., “Prognostic Value of HALP Score in Patients With Valvular Heart Disease: Insights From a Large‐Scale Chinese Cohort,” BMC Cardiovascular Disorders 25 (2025): 178, 10.1186/s12872-025-04968-2.40082803

[10] J. Tang , X. Zhao , W. Li , et al., “Hematological and Nutritional Indices as Predictors of Outcome in Rheumatic Mitral Valve Disease,” Circulation: Cardiovascular Imaging 17, no. 2 (2024): e016302, 10.1161/CIRCIMAGING.123.016302. 39405388

[11] M. K. Akboğa , U. Canpolat , Ç. Yayla , et al., “Neutrophil‐to‐Lymphocyte Ratio Is Increased in Patients With Rheumatic Mitral Valve Stenosis,” Anatolian Journal of Cardiology 14, no. 2 (2014): 130–134, 10.5152/akd.2013.4643. PMC5779174 25430404

[12] A. S. Rambe and R. M. Putri , “Neutrophil‐to‐Lymphocyte Ratio as a Predictor of Severity in Patients With Rheumatic Mitral Valve Stenosis,” Journal of Society Medicine 6, no. 1 (2023): 30–36.

[13] M. B. Karataş , G. İpek , T. Onuk , et al., “Relationship Between Platelet‐to‐Lymphocyte Ratio and Rheumatic Heart Disease,” Eurasian Journal of Medical Investigation 1, no. 2 (2017): 53–58, 10.14744/ejmi.2017.37275.

