# Correction to “Prognostic Value of HALP Score in Rheumatic Mitral Stenosis: A Long‐Term Follow‐up Study”

**DOI:** 10.1002/clc.70361

**Published:** 2026-06-05

Kaya AF, Ayhan G, Can V, Özbek E, Dursun L, Kümet Ö. Prognostic value of HALP score in rheumatic mitral stenosis: a long‐term follow‐up study. *Clinical Cardiology* 49, no. 4 (2026): e70287. doi:10.1002/clc.70287

In the published version of this article, the study population was incorrectly reported as *N* = 169 in several locations, including the abstract, main text, and table headers. The correct number of patients analyzed after applying the exclusion criteria is *N* = 127.

All statistical analyses (including ROC curve analysis, Kaplan–Meier survival analysis, and Cox regression) were performed using the correct data set (*N* = 127); therefore, the reported results, statistical values, and conclusions of the study are unchanged.

In addition, Table 1 has been corrected to remove an erroneously listed “Chronic Kidney Disease” row (patients with CKD had been excluded), and the cardiac rhythm counts have been corrected so that they sum to 127 (39 atrial fibrillation, 88 sinus rhythm). Minor formatting and unit errors in Tables 2 and 3 have also been fixed. The corrected tables are as follows:

**Table 1:** Demographic and clinical characteristics of the study population.

Variable	All patients (*n* = 127)
Sex, *n* (%)	
Female	104 (81.9)
Male	23 (18.1)
Age, years	41 ± 11.8
Hyperlipidemia, *n* (%)	17 (13.4)
Diabetes mellitus, *n* (%)	13 (10.2)
Hypertension, *n* (%)	18 (14.2)
Cardiac rhythm, *n* (%)	
Atrial fibrillation	39 (30.7)
Sinus rhythm	88 (69.3)
Smoking, *n* (%)	37 (29.1)
MACCE, *n* (%)	
None	106 (83.5)
Myocardial infarction	1 (0.8)
Cerebrovascular event	3 (2.4)
Death	17 (13.4)
Percutaneous mitral balloon valvuloplasty, *n* (%)	35 (27.6)
Surgery, *n* (%)	23 (18.1)
Follow‐up duration, months	124 (96–148)
Medications, *n* (%)	
Beta‐blockers	121 (95.3)
ACE inhibitors/ARBs	13 (11.0)
Mineralocorticoid receptor antagonists	15 (11.8)
Oral anticoagulants	21 (16.5)
Diuretics	68 (53.5)

Abbreviations: ACE, angiotensin‐converting enzyme; ARB, angiotensin receptor blocker; MACCE, major adverse cardiac and cerebrovascular events.

**Table 2:** Laboratory findings.

Laboratory findings	All patients (*n* = 127)
Glucose (mg/dL)	101 (91–115)
Urea (mg/dL)	27 (21–34)
Creatinine (mg/dL)	0.7 (0.6–0.8)
Sodium (mEq/L)	137 (135–139)
Potassium (mEq/L)	4.2 (4.0–4.5)
Calcium (mg/dL)	9.0 (8.5–9.5)
C‐reactive protein (mg/dL)	0.8 (0.4–1.2)
LDH (U/L)	276.9 ± 104.4
Platelet (10^3^/mm^3^)	255.6 ± 93.7
MPV (fL)	8.9 ± 1.8
Albumin (g/dL)	3.6 (3.3–4.0)
Hemoglobin (g/dL)	12.5 ± 1.7
Hematocrit (%)	38.4 ± 5.0
MCV (fL)	84.0 (80.0–87.0)
MCH (pg)	28.0 (26.0–29.0)
MCHC (g/dL)	33.0 (32.0–34.0)
RDW (%)	13.8 ± 2.4
AST (IU/L)	22.0 (17.0–28.0)
ALT (IU/L)	17.0 (12.0–25.0)
WBC (×10^3^/µL)	9.0 (7.5–10.1)
RBC (×10^6^/µL)	4.5 ± 0.6
Neutrophils (×10^9^/L)	5.6 (4.4–7.4)
Lymphocytes (×10^9^/L)	2.1 (1.6–2.6)
Monocytes (×10^9^/L)	0.5 (0.4–0.7)
Basophils (%)	0.07 (0.05–0.10)
Eosinophils (%)	0.09 (0.06–0.17)

*Note:* Values are presented as median (Interquartile range) or mean ± standard deviation, as appropriate.

**Table 3:** Echocardiographic parameters.

Echocardiographic parameters	All patients (*n* = 127)
Ejection fraction (%)	60 (60–60)
Left atrial diameter (mm)	46 (43–51)
Interventricular septum (mm)	10.0 (9.0–11.0)
Left ventricular end‐diastolic diameter (mm)	46.0 ± 4.7
Mitral valve area (PHT, cm^2^)	1.30 (1.0–1.4)
Mitral valve area (Planimetry, cm^2^)	1.30 (1.0–1.4)
Mean gradient (mmHg)	10.0 (8.0–13.0)
Maximum gradient (mmHg)	20.0 (16.0–25.0)
Mitral stenosis severity—None	0 (0)
Mitral stenosis severity—Mild	0 (0)
Mitral stenosis severity—Moderate	87 (68.5)
Mitral stenosis severity—Severe	40 (31.5)
Two‐valve involvement	57 (44.9)
Aortic regurgitation—None	70 (55.1)
Aortic regurgitation—Mild	42 (33.1)
Aortic regurgitation—Moderate	14 (11.0)
Aortic regurgitation—Severe	1 (0.8)
Mitral regurgitation—None	49 (38.6)
Mitral regurgitation—Mild	51 (40.2)
Mitral regurgitation—Moderate	25 (19.7)
Mitral regurgitation—Severe	2 (1.6)
Tricuspid regurgitation—None	11 (8.7)
Tricuspid regurgitation—Mild	80 (63.0)
Tricuspid regurgitation—Moderate	29 (22.8)
Tricuspid regurgitation—Severe	7 (5.5)
Systolic pulmonary artery pressure (mmHg)	40.0 (30.0–50.0)

*Note:* Values are presented as median (Interquartile range) or mean ± standard deviation, as appropriate.

Abbreviation: PHT, pressure half‐time.

We apologize for these errors.

